# Is the Salivary Gland Associated with Honey Bee Recognition Compounds in Worker Honey Bees (*Apis mellifera*)?

**DOI:** 10.1007/s10886-018-0975-8

**Published:** 2018-06-07

**Authors:** Stephen J. Martin, Maria E. Correia-Oliveira, Sue Shemilt, Falko P. Drijfhout

**Affiliations:** 1 School of Environment and Life Sciences, The University of Salford, Manchester, M5 4WT UK; 2 Insecta Research Group, Center of Agrarian, Environmental and Biological Sciences, Federal University of the Reconcavo of Bahia, Rua Rui Barbosa, 710 - Centro, Cruz das Almas, BA 44380-000 Brazil; 3 Chemical Ecology Group, School of Chemical and Physical Sciences, Lennard-Jones Laboratory, Keele University, Keele, ST5 5BG UK

**Keywords:** *Apis mellifera*, Cuticular profiles, Cephalic salivary gland, Mandibular gland, Exocrine glands, Post-pharyngeal gland, Sociobiology

## Abstract

Cuticular hydrocarbons (CHCs) function as recognition compounds with the best evidence coming from social insects such as ants and honey bees. The major exocrine gland involved in hydrocarbon storage in ants is the post-pharyngeal gland (PPG) in the head. It is still not clearly understood where CHCs are stored in the honey bee. The aim of this study was to investigate the hydrocarbons and esters found in five major worker honey bee (*Apis mellifera*) exocrine glands, at three different developmental stages (newly emerged, nurse, and forager) using a high temperature GC analysis. We found the hypopharyngeal gland contained no hydrocarbons nor esters, and the thoracic salivary and mandibular glands only contained trace amounts of *n*-alkanes. However, the cephalic salivary gland (CSG) contained the greatest number and highest quantity of hydrocarbons relative to the five other glands with many of the hydrocarbons also found in the Dufour’s gland, but at much lower levels. We discovered a series of oleic acid wax esters that lay beyond the detection of standard GC columns. As a bee’s activities changed, as it ages, the types of compounds detected in the CSG also changed. For example, newly emerged bees have predominately C_19_-C_23_*n*-alkanes, alkenes and methyl-branched compounds, whereas the nurses’ CSG had predominately C_31:1_ and C_33:1_ alkene isomers, which are replaced by a series of oleic acid wax esters in foragers. These changes in the CSG were mirrored by corresponding changes in the adults’ CHCs profile. This indicates that the CSG may have a parallel function to the PPG found in ants acting as a major storage gland of CHCs. As the CSG duct opens into the buccal cavity the hydrocarbons can be worked into the comb wax and could help explain the role of comb wax in nestmate recognition experiments.

## Introduction

Pheromones are involved in intraspecific chemical communication (Wyatt 2013), and the honey bee (*Apis mellifera*) has long been the subject of chemical ecology studies (Breed et al. 2015; Free 1987). However, the glands associated with compounds used in nestmate recognition in honey bees remain elusive. This search is difficult since nestmate cues can arise from both within the colony, and from the environment (Kalmus and Ribbands 1952). For example, Downs and Ratnieks (1999) found no evidence that honey bee guards used heritable cues; instead, guards appear to rely exclusively on environmental cues to distinguish nestmates from non-nestmates. However, nestmate cues can be produced by the individual, and thus must be under genetic control (Breed 1983; Page Jr et al. 1991). A further factor is that the wax used to build the colony is both produced and manipulated by the bees, which means it may be a medium into which recognition cues are transferred (Breed et al. 1998). Therefore, Breed et al. (1998) stated that no single factor is responsible for nestmate recognition in honey bees; rather, all three factors (genetically determined cuticular signatures, exposure to comb wax, and environmental cues e.g. floral cues) seem to work together.

In ants, the post-pharyngeal gland (PPG) found in the head can absorb, store, and metabolize its own lipids (Decio et al. 2016), and the hydrocarbon profile (ratio of compounds) of the PPG is very similar to that of the cuticle (Bagnères and Morgan 1991; Kaib et al. 2000). The PPG may also act as a mixing ‘*gestalt*’ organ (Soroker et al. 1994), since cuticular hydrocarbons (CHCs) can be transferred to an individual’s PPG by nestmates via the sharing of secretions (trophallaxis) or grooming (Meskali et al. 1995; Morgan 2010). Thus, any genetic (CHCs) or environmental differences between individuals can be smoothed out, producing a single unified nestmate odour.

Work, predominantly on social insects, has shown that each species of insect has a unique CHC profile (Kather and Martin 2015), which is remarkably stable over large geographical areas (Guillem et al. 2016). In ants, qualitative differences in these unique species profiles indicates that the CHCs can also contain a nestmate signal (Martin et al. 2008; van Zweden and d’Ettorre 2010). Several studies have indicated the importance of CHCs in nestmate recognition in honey bees. For example, newly emerged bees, which have fewer hydrocarbons in their cuticle, are accepted more readily into an unrelated colony, while the removal of hydrocarbons from older bees improves their acceptance (Breed et al. 2004). Further studies indicated that a sub-set of hydrocarbons, the long-chained alkenes, maybe involved in honey bee nestmate recognition (Châline et al. 2005; Dani et al. 2005; Pradella et al. 2015). However, the location where hydrocarbons, especially alkenes, are stored prior to release onto the cuticle remains unclear.

**Fig. 1:**
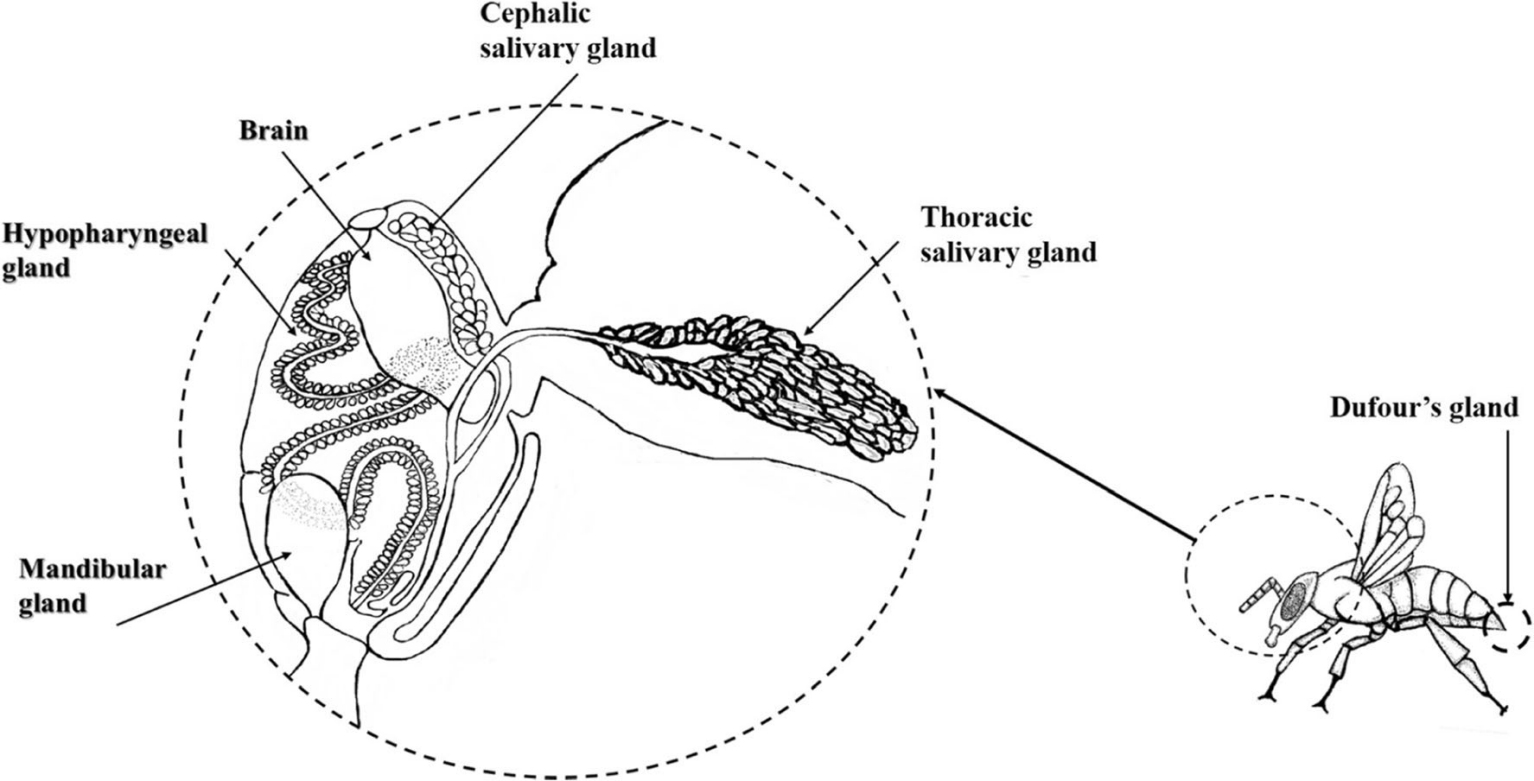
The position of the five glands, three in the head, one in the thorax, and one in the gaster, evaluated in this study (adapted from Tofilski 2012)

The five major glands studied (mandibular [MG], hypopharyngeal [HG], cephalic salivary [CSG], thoracic salivary [TSG], and Dufour’s gland [DG]) (Fig. 1) are all vital to an adult worker performing its various tasks both inside and outside the hive successfully (Duffield et al. 1984; Katzav-Gozansky et al. 2001; Poiani and Cruz-Landim 2017). The MG, HG, CSG, and TSG are all paired glands whose ducts open into the buccal cavity (Snodgrass 1956). The primary role of the HG is providing protein rich secretions to feed the larvae during the nursing phase (Crailsheim and Stolberg 1989; Huang et al. 1989), and in foragers these glands secrete enzymes that aid the processing of honey (Simpson 1960). The MG initially secretes fatty acids, again used for larval nutrition (Plettner et al. 1997). They then switch to the secretion of ‘forage-marking’ (Vallet et al. 1991) and alarm pheromones such as 2-heptanone in foragers. In worker honey bees, the salivary gland develops into two glands with the CSG found in the head and the TSG in the thorax. Simpson (1960) noted the secretion of the TSG was aqueous, containing water soluble digestive enzymes, whereas the CSG produced an oily secretion that helps with wax manipulation. However, Katzav-Gozansky et al. (2001) detected a series of *n*-alkanes in both the TSG and CSG, which partly conflicting with what Simpson (1960) found. Recently, and based on a single colony, Poiani and Cruz-Landim (2017) suggested that the CSG secretion may be used to replenish the CHCs. In contrast the TSG secretions are important for honey maturation (Maurizio 1975), possibly contain pheromones (Maurizio 1975) and help moisten pollen and wax (Simpson 1963). The DG is associated with the sting apparatus and although well studied in queens (Niño et al. 2013) and workers in queen-less colonies, its role in workers from queenright colonies is not well defined (Mitra 2013). It is known to produce hydrocarbons (Katzav-Gozansky et al. 2001) that are used for waterproofing the cuticle and possibly involved in chemical communication (Abdalla and Cruz-Landim 2001).

The aim of this study was to investigate the presence or absence of hydrocarbons contained within five honey bee exocrine glands, at three different key points in the adult honey bee worker’s life (newly emerged, nurse, and foraging phases). This data was then compared with the CHC profiles from workers doing the same tasks (Kather et al. 2011). This will determine which gland is acting as a reservoir for hydrocarbons that are detected on the surface of the honey bee i.e. CHCs, and will also help determine whether the role of the glands changed as the tasks of the bee changed.

## Methods and Materials

### Samples Collection and Preparation

All studies were conducted during the summer of 2014 using four honey bee colonies maintained at the University of Salford apiary Manchester, UK. Worker bees of known age were obtained by removing a frame containing mature pupae and placing it into a temperature cabinet controlled at 30 °C ± 5 °C and RH 60% ± 2%. The thorax of approximately 500 newly born workers per colony were marked with a non-toxic Uniposca® pen before being returning to their natal colony. Thereafter, from each of the four colonies, 50 workers were sampled when they emerged (newly emerged bees [day 0]). After 10 days bees were collected from the surface of a frame whilst feeding larva (nurse bees), and after 30 days bees were collected returning to the hive bearing pollen (forager bees). Since hydrocarbons are known to be stable under a wide range of storage conditions (Martin et al. 2009), all collected bees were stored at −20 °C until their glands were removed. Only workers were sampled since CSG glands are poorly developed in male honey bees (Poiani and Cruz-Landim 2010).

### Gland Extraction

The five glands (Fig. 1) were dissected out in water using fine tweezers under a Leica binocular microscope at ×25 magnification. The head was separated from the thorax using micro scissors and the TSG extracted from the thorax using fine tweezers. From the head, the buccal apparatus was removed by creating a hole near the mandibles, which was then extended into the ventral region to allow the removal of the MG. The exoskeleton in the dorsal region of the head was removed allowing the HG and CSG to be removed. By removing the stinger, the DG could easily be extracted.

### Chemical Analysis

Each sample was a pool of 10 glands to ensure a clear chemical signal. For each colony, five replicates of each age group (0, 10, and 30 days) were prepared. This was replicated across the four colonies giving a total of 300 samples to be analysed (four colonies x five replicates x three age groups x five glands), from 600 bee dissections. Each sample was immersed in 30 μl of high-performance liquid chromatography (HPLC) grade *n*-hexane and evaporated to dryness at room temperature before being stored at −20 °C. Immediately before analysis samples were re-suspended in 30 μl HPLC grade *n*-hexane and analysed on an Agilent 7890-GC (equipped with a Vf-5ht UltiMetal column; length: 30 m; ID: 0.25 mm; film thickness; 0.1 μm) connected to an Agilent 5975-MSD (quadrupole mass spectrometer with 70-eV electron impact ionization). Samples were injected in splitless mode with the injection port at 325 °C and the MS in scan mode. The oven temperature programme was 70 °C (held for 1 min), 40 °C min^−1^ to 200 °C, 4 °C min^−1^ to 250 °C and finally 25°Cmin^−1^ to 350 °C with a final five-minute hold. The carrier gas of helium was used at a constant flow rate of 1.0 ml min^−1^. Compounds were identified using standard MS databases, diagnostic ions, and Kovats indices. This method allowed us to detect hydrocarbons and esters up to chain lengths of forty-two carbons (*n*-C_42_). The presence of oleic acid in samples was variable, potentially due to contamination from other body tissues, so was excluded from the analysis. Each peak was transformed into a relative proportion based on the total amount of hydrocarbons (C_19_ to C_33_) plus wax esters, if present. Any compound which was consistently less than 1% across all three age groups was excluded from further analysis and recorded as trace (Table 1). An internal standard was not used so all comparisons are relative.

**Table 1:** The percentage of hydrocarbons and oleic acid wax esters detected in four major glands in three different age groups of honey bees

			Cephalic salivary gland			Thoracic salivary			Dufour’s gland			Mandibular gland			Cuticular surface (Kather et al. 2011)		
Peak #	Hydrocarbon abbreviation	Ret time	Day 0	Day 10	Day 30	Day0	Day 10	Day 30	Day 0	Day 10	Day 30	Day 0	Day 10	Day 30	Day 0	Day 10	Day 20
1	C_19:1_	6.8	2 ± 1	t	t	–	–	–	–	–	–	–	–	–	–	–	–
2	*n*-C_19_	6.9	2 ± 1	t	t	–	–	–	–	–	–	–	–	–	–	–	–
3	C_21:1_	8.2	t	t	1 ± 1	–	–	–	–	–	–	–	–	–	–	–	–
4	*n*-C_21_	8.4	**16 ± 5**	4 ± 3	t	–	–	–	6.9 ± 5.4	–	4.8 ± 2.6	**10 ± 3.8**	–	–	2.5 ± 2.1	–	–
5	C_23:1_	10.2	9 ± 3	3 ± 2	1 ± 1	–	–	–	–	–	–	5.4 ± 3.9	–	–	0.9 ± 0.8	0.9 ± 1.1	5 ± 1.9
6	*n*-C_23_	10.6	**29 ± 9**	7 ± 3	**16 ± 9**	–	–	–	**18 ± 15**	6.7 ± 4.2	4 ± 3.3.	**15 ± 8.2**	–	–	**19 ± 6.7**	8.6 ± 5.3	**25 ± 6.3**
7	C_23_-Me3	11.6	**–**	–	**–**	–	–	–	**–**	2.4 ± 4.4	–	**–**	–	–	**–**	–	**–**
8	C_25:2_	12.5	3 ± 1	t	t	–	–	–	–	–	–	–	–	–	–	–	–
9	C_25:1_	12.8	4 ± 2	4 ± 2	1 ± 1	–	–	–	–	–	–	5.8 ± 2.5	–	–	1.7 ± 1.3	1.3 ± 1.3	5.5 ± 1.6
10	*n*-C_25_	13.2	6 ± 3	6 ± 3	**18 ± 9**	**100**	**97 ± 10**	**100**	**19 ± 14**	**20 ± 12**	**13 ± 4.7**	**13 ± 5.1**	**63 ± 19**	**81 ± 14**	**10 ± 1.7**	9.3 ± 3.4	**18 ± 3.4**
11	C_25_ Me9, 11, 13	13.6	2 ± 2	t	t	–	–	–	5.3 ± 7.1	–	–	3.3 ± 2.8	–	–	2.8 ± 0.8	0.4 ± 0.6	0.4 ± 0.4
12	C_27:2_	15.3	t	t	t	–	–	–	–	–	–	–	–	–	0.2 ± 0.1	0.7 ± 0.5	1.5 ± 0.5
13	C_27:1_	15.7	t	2 ± 1	1 ± 1	–	–	–	–	–	–	–	–	–	t	0.1 ± 0.3	1.3 ± 0.5
14	*n*-C_27_	16.1	5 ± 2	6 ± 3	7 ± 5	–	3 ± 10	–	**15** ± 9.5	**19** ± 7	**17** ± 4.8	**13 ± 3.3**	**15 ± 7.4**	**19 ± 14**	**27 ± 3.5**	**19 ± 4.1**	**16 ± 4.4**
15	C_27_ Me9, 11, 13	16.6	5 ± 1	1 ± 0	t	–	–	–	8.1 ± 8.4	–		**12 ± 3.9**	**–**	**–**	**14 ± 3.7**	1.8 ± 0.9	0.7 ± 0.5
16	C_27_-Me3	17.1	–	–	–	–	–	–	–	–	3.3 ± 2.6	–			–	–	–
17	C_29:1_	18.2	t	2 ± 1	1 ± 1	–	–	–	–	–	–	–	**–**	**–**	0.4 ± 1.0	2.6 ± 0.9	2 ± 1
18	*n*-C_29_	18.4	1 ± 1	3 ± 2	2 ± 2	–	–	–	8.2 ± 6.6	**23 ± 7**	**21 ± 5**	5.6 ± 3	**–**	**–**	4.8 ± 2.6	**11 ± 2.1**	5.8 ± 2.4
19	C_29_ Me9, 11, 13	18.6	3 ± 1	t	t	–	–	–	7.4 ± 8.5	–	–	9.7 ± 2.9	**–**	**–**	8.8 ± 3.0	1.2 ± 0.6	0.8 ± 1.5
20	C_31:1_ (2 isomers)	19.4	2 ± 2	**11 ± 6**	2 ± 2	–	–	–	1.8 ± 1.9	4.1 ± 4	2.4 ± 5.3	2.8 ± 2.1	**–**	**–**	1.3 ± 2.6	**19 ± 5.9**	8.4 ± 2.6
21	*n*-C_31_	19.6	t	2 ± 1	1 ± 2	–	–	–	3 ± 5.4	**18 ± 6**	**17 ± 6**	1.6 ± 1.7	**–**	**–**	0.8 ± 1.2	6.3 ± 2.1	2.3 ± 1.1
22	C_31_ Me9, 11, 13	19.7	t	1 ± 3	t	–	–	–	2.9 ± 3.5	–	–	3.4 ± 1.4	**–**	**–**	3.1 ± 1.3	0.1 ± 0.3	0.2 ± 0.3
23	C_33:1_ (3 isomers)	20.3	2 ± 3	**19 ± 11**	2 ± 2	–	–	–	0.7 ± 1.4	3.4 ± 3.8	1.1 ± 1.2	–	**22 ± 14**	–	1.1 ± 2.6	**18 ± 10**	5.5 ± 3.3
24	*n*-C_33_	20.4	–	**–**	–	–	–	–	–	1.4 ± 0.9	1.6 ± 0.6	–	**–**	**–**	0.5 ± 1.5	0.1 ± 0.2	0.4 ± 0.3
25	C_33_-Me9, 11, 13	20.5	–	**–**	–	–	–	–	0.9 ± 1.5	**–**	**–**	–	**–**	**–**	0.4 ± 0.3	**–**	0.3 ± 0.4
26	Oleic acid octadecyl ester	21.7	2 ± 3	7 ± 8	**13 ± 9**	–	–	–	0.1 ± 0.2	0.9 ± 1.2	1.6 ± 0.6	–	**–**	**–**	–	–	–
27	Oleic acid eicosyl ester	22.3	2 ± 4	**15 ± 15**	**28 ± 13**	–	–	–	0.7 ± 1.5	0.9 ± 1	8.3 ± 3.9	–	**–**	**–**	–	–	–
28	Oleic acid tetracosyl ester	23.0	3 ± 7	3 ± 2	3 ± 1	–	–	–	–	–	–	–	**–**	**–**	–	–	–
29	Unidentified wax ester	23.5	t	t	2 ± 2	–	–	–	–	0.6 ± 0.9	1.5 ± 0.7	–	**–**	**–**	–	–	–
30	Unidentified wax ester	23.9	t	t	2 ± 1	–	–	–	0.9 ± 1	–	2.2 ± 0.9	–	**–**	**–**	–	–	–

Compounds are ordered by retention time (Ret). Each value is the average (±SD) of between 14 and 20 samples per age/task group. These are compared with the cuticular hydrocarbon profiles extracted from whole body washes of between 33 to 44 workers of each age/task group (Kather et al. 2011). All values greater than 10% are presented in bold for clarity, t = trace proportion, i.e. detectable but too small to be reliably integrated

### Statistical Analysis

The raw data was arcsine square root transformed to reduce the range of data values, and non-metric Multidimensional Scaling (NMDS) performed using Euclidean distances in SPSS v23. Goodness of fit was measured using the Normalized raw stress. The lower the stress value, the better the data are represented, with values of <0.05 considered excellent, and where values of >0.2 are considered a poor representation of the data and should be interpreted with caution. NMDS does not rely on the assumptions of normality of multidimensional data, thus having the advantage of omitting any distributional assumptions required for other methods (McCune and Grace 2002). Moreover, the pre-assumed hypothesis-driven groupings such as those used in discriminant analysis, which can bias the final result, as can minor compounds (Martin and Drijfhout 2009). Also, if the number of variables is sufficiently large, which is often the case, the canonical variates separate the groups regardless of the actual group distributions (Mitteroecker and Bookstein 2011). The CHC profiles from workers (Kather et al. 2011) and CSG (this study) came from the same honeybee colonies, but the CHC profiles were analysed using a standard GC column, so before the datasets were compared the high temperature oleic acid waxy esters were removed from the data-set (see Fig. 3b).

## Results

In the TSG trace amounts of *n*-pentacosane (*n*-C_25_) were detected, irrespective of age group and no other non-polar compounds were seen. As expected no hydrocarbons were detected in the HG, while the MG of new-born bees contained only small amounts of the *n*-alkanes and alkenes typically found in the CSG and DG, which became trace amounts of *n*-C_25_ and *n*-heptacosane (*n*-C_27_) in older age groups (Table 1). The DG contained predominantly (70–88%) odd chained *n*-alkanes (*n*-heneicosane [*n*-C_21_] to *n*-hentriacontane [*n*-C_31_]), with small proportions of alkenes (3–8%), whereas the methyl branched-alkanes occur predominantly in newly emerged bees, and the oleic acid wax esters were found mainly in the foragers (Table 1).

**Fig. 2:**
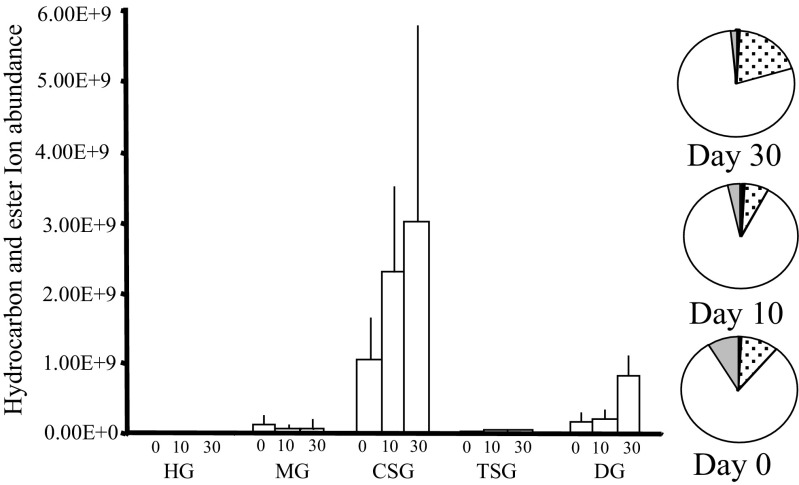
The relative amount, based on total ion counts of CHCs and oleic acid wax esters, in the five exocrine glands (HG = hypopharyngeal, MG = mandibular, CSG = cephalic salivary, TSG = thoracic salivary, and DG = Dufour’s gland), at the three different honey bee ages; day 0 = newly emerged, day 10 = nurses, and day 30 = foragers. The error bars represent +1 SD. The pie charts indicate the proportions of the CHC and oleic acid wax esters at each age (CSG = clear, MG = grey, DG = dotted and MG = black)

**Fig. 3:**
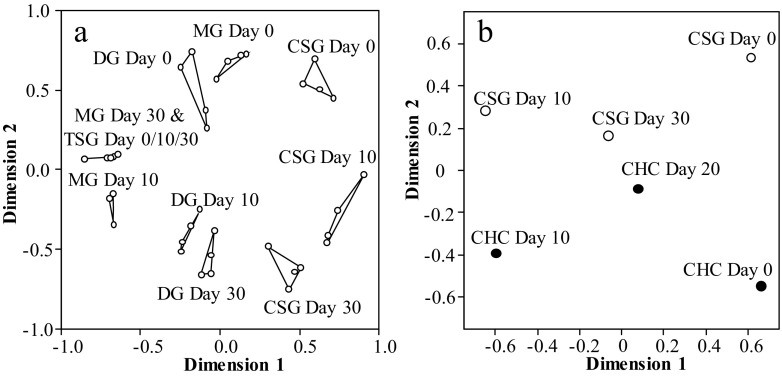
**a** NMDS analysis of the four glands contain different hydrocarbons that change as the bees aged (see Table 1). Each dot represents the mean profile for each colony. **b** NMDS analysis indicates the similarity between the CSG and CHC profile for each age group (dimension 1). Each dot represents the mean chemical profile (excluding esters) for each group across all samples in each of the four colonies. The Normalized raw stress values were a) 0.05184 and b) 0.01813, indicating good representation of the data

**Fig. 4:**
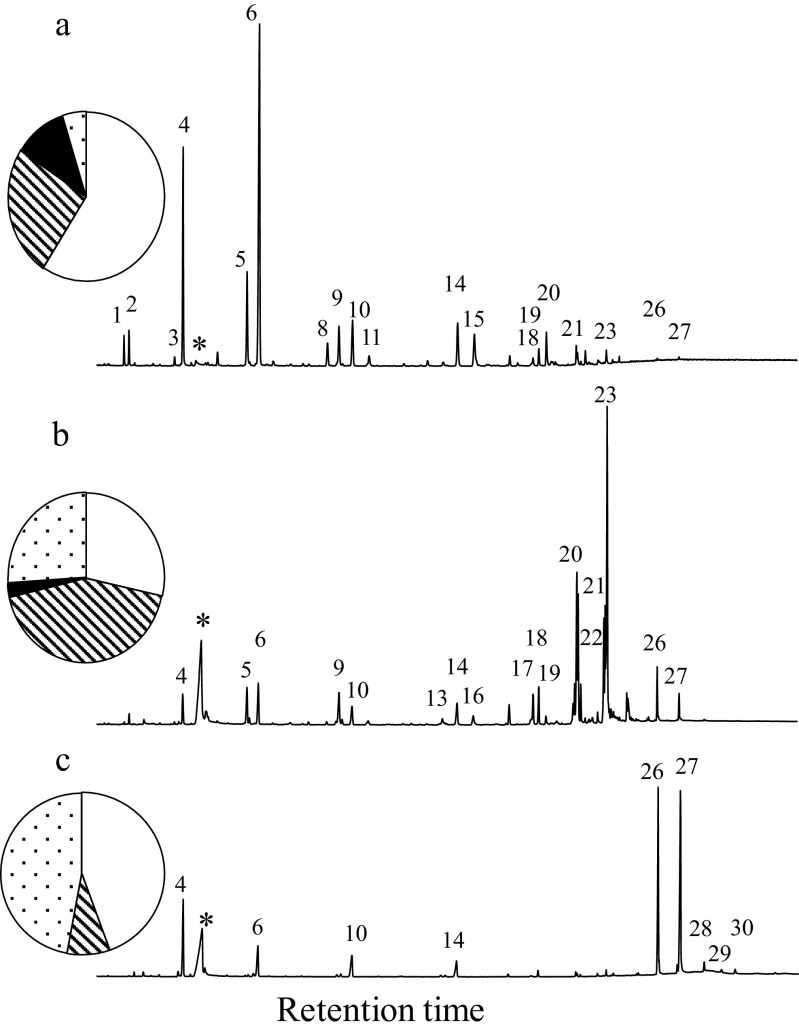
Typical Total Ion Chromatograms of the cephalic salivary gland from (**a**) newly emerged bees, (**b**) nurse bees, and (**c**) foragers. The numbers indicate the compounds presented in Table 1. The pie diagrams indicate the proportion of *n*-alkanes (clear), alkenes (lines), methyl branched-alkanes (black) and oleic acid wax esters (dots). * = Oleic acid (C_18_)

**Fig. 5:**
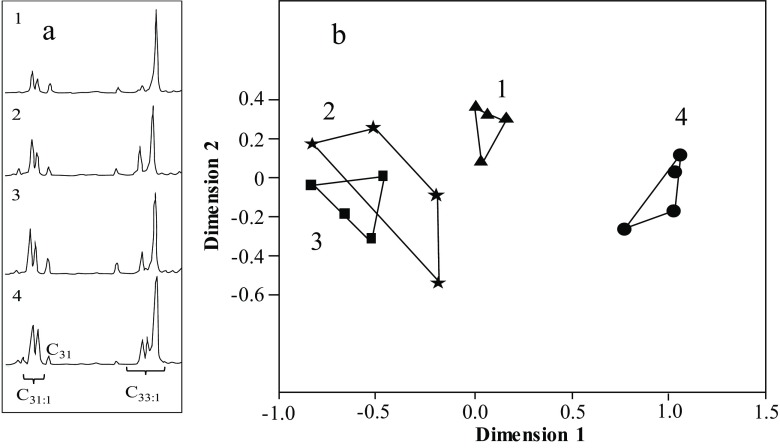
**a** The colony specific differences in the C_31:1_ and C_33:1_ isomers ratios between the four colonies detected in the cephalic salivary glands at day 10. Colonies are sometimes distinct (1, 4 and 2) or less distinct (2 and 3). **b** These observations were supported by an NMDS analysis with a normalized raw stress value of 0.00779. Each point represents a pooled 10-day CSG sample from each of the four colonies

The CSG contained, relative to the other glands, a large quantity of *n*-alkanes, alkenes, methyl branched-alkanes, and oleic acid wax esters (Table 1; Fig. 2). The proportions of these compounds changed dramatically as the bees aged (Table 1; Fig. 3a) and mirrored the changes seen in the adult CHC profiles (Table 1: Fig. 3b). In newly emerged bees (day 0) 10% of the CSG consisted of methyl branched-alkanes, which dropped to just 2% after 10 days and to trace amounts at 30 days. The dominant hydrocarbons (47%) in newly emerged workers were the three *n*-alkanes (*n*-C_19_, *n*-C_21,_ and *n*-C_23_), these were replaced by C_31:1_ and C_33:1_ alkene isomers (30%) after 10 days, before the appearance of the oleic acid wax esters (48%) in the 30-day old bees (foragers) (Fig. 4; Table 1). This changing pattern was mirrored in the DG (Table 1; Fig. 3a). In the CSG gland the C_31:1_ and C_33:1_ alkene isomers were present in all age groups but were a major part of the profile in 10-day old bees (nurses). At this time colony-specific patterns in the C_31:1_ and C_33:1_ isomer ratios were seen in all colonies. In other words, there was little within colony variation of the isomer pattern for all four colonies, whilst between colony difference could be detected (Fig. 5). These patterns cannot be detected in day 0 and day 30 bees due to the small amounts of alkenes detected. However, such colony-specific patterns were not seen in any other group of compounds.

## Discussion

This study found that, among the five major exocrine glands studied, the CSG was the only one to contain large amounts of CHCs. The production, or storage, of lipids in the CSG, and not the TSG, is supported by evidence from both physiological (Poiani and Cruz-Landim 2010) and proteomic (Fujita et al. 2010) studies. The CHCs detected in the CSG are typical of those reported on the surface of adult honey bees (Table 1, Kather et al. 2011; Pradella et al. 2015), thus supporting the findings of Poiani and Cruz-Landim (2017). Across a wide range of Hymenoptera the DG also contains hydrocarbons found on their cuticle. These include bumblebees (Oldham et al. 1994), social *Polistes* (Dani et al. 1996) and *Ropalidia* wasps (Mitra and Gadagkar 2014), as well as solitary wasps (Howard and Baker 2003) and solitary bees (Pitts-Singer et al. 2017). Interestingly, in the stingless bee *Melipona bicolor* the DG has been lost in the workers (Abdalla et al. 2004) but remains in the queen and contains hydrocarbons and esters, as found in many social Hymenoptera queens (Le Conte and Hefetz 2008).

In this study the MG contained a range of hydrocarbons in newly emerged bees that became reduced to only *n*-pentacosane and *n*-heptacosane as the bees aged, with tritriacontene (C_33:1_) appearing in the nurses (Table 1). However, in the stingless bee *Melipona quadrifasciata, n*-alkanes and alkenes found in the MG persist throughout the entire adult life and are similar to the adults CHCs (Cruz-Landim et al. 2012). Therefore, the functions of the CSG and MG may be interchangeable in different genera of bees.

The predominance of alkene isomers C_31:1_ and C_33:1_ found in the CSG, especially in nurse bees, suggests a role in sequestering recognition compounds. It has been suggested that these alkenes serve as nestmate recognition compounds in honey bees (Châline et al. 2005; Dani et al. 2005; Pradella et al. 2015). Furthermore, a large diversification of alkene isomers are uniquely found among bees (Kather and Martin 2015a) indicating that these play an important role in chemical communication in this group. Interestingly, a similar pattern of *n*-alkanes, alkenes, and oleic acid wax esters found in the CSG are also present in comb wax (Aichholz and Lorbeer 2000; Fröhlich et al. 2000; Waś et al. 2014). Of the three age groups studied, only the nurse bees have active wax glands, which reach their maximum size in 5 to 15-day old workers (Blomquist et al. 1980). The wax scale produced by the nurse bee is manipulated into brood cells by adding a frothy saliva substance that increases lipolytic activity, helping to change the waxes physical structure (Kurstjens et al. 1985). During this time compounds from the CSG could be added to the comb wax. This may help explain why cues gained from comb wax can affect a honey bees’ level of aggression towards its nestmates (Breed et al. 1998; Downs and Ratnieks 1999). In male honey bees the CSG is poorly developed and regress with age (Poiani and Cruz-Landim 2010). However, in male Bumblebees (*Bombus* spp) the CSG is well developed and contains species-specific sexual marking pheromones (Šobotnik et al. 2008).

This study and a previous one (Poiani and Cruz-Landim 2017) both found similar age-related changes in the hydrocarbons detected in the CSG despite being conducted in Brazil and UK and on different sub-species of bee (Africanised and Buckfast). This suggests these patterns of change are widespread across *A. mellifera*. That is, the methyl branched-alkanes associated with newly emerged bees disappear from the CSG and CHCs after 10 days. In contrast, alkene production peaks during the nurse phase, potentially allowing them to be incorporated into the wax-comb. While the CSG of foragers is dominated by several long (>C_32_) waxy esters. Similar heavy (>C_35_) waxy esters have been detected on the surface of 11 species of insects from nine genera of Hymenoptera using high temperature GC-MS (Sutton et al. 2013) and could be ubiquitous in social insects, acting as waterproofing compounds (Nelson et al. 2001), although they could also play a role in communication. For example, Amsalem et al. (2009) found a series of C_24_-C_28_ wax esters in the DG of *Bombus terrestris* workers that did not lay eggs and suggested they signal a worker’s functional sterility to other members of the colony. Furthermore, the honey bee brood pheromone is composed of a series of C_17_-C_20_ fatty acid esters that act as a primer pheromone (Le Conte et al. 2001). These esters are produced in the salivary gland of the larvae prior to it bifurcating into the CSG and TSG during the pupal stage.

This study indicated that the CSG in honey bees has a parallel function to the PPG in ants and wasps (Herznera et al. 2011), functioning as a reservoir for hydrocarbons that are also present on the surface of honey bees. In addition, the CSG contains a group of oleic acid wax esters whose function needs to be elucidated in the future.

## References

[CR1] Abdalla FC, Cruz-Landim C (2001). Behavioral responses evoked in honey bee workers by Dufour gland extracts (Hymenoptera: Apidae). Sociobiology.

[CR2] Abdalla FC, Graeme RJ, Morgan D, Cruz-Landim C (2004). Chemical composition of the Dufour gland secretion in queens of *Melipona bicolor* (Hymenoptera, *Meliponini*). J Braz Chem Soc.

[CR3] Aichholz R, Lorbeer E (2000). Investigation of comb wax of honey bees with high-temperature gas chromatography and high-temperature gas chromatography-chemical ionization mass spectrometry. II. High-temperature gas chromatography chemical ionization mass spectrometry. J Chromatogr A.

[CR4] Amsalem E, Twele R, Francke W, Hefetz A (2009). Reproductive competition in the bumble-bee *Bombus terrestris:* do workers advertise sterility?. Proc R Soc B.

[CR5] Bagnères AG, Morgan ED (1991). The postpharyngeal gland and the cuticle of *Formicidae* contain the same characteristic hydrocarbons. Experientia.

[CR6] Blomquist GJ, Chu AJ, Remaley S (1980). Biosynthesis of wax in the honeybee, *Apis mellifera* L. Insect Biochem Mol Biol.

[CR7] Breed MD (1983). Nestmate recognition in honey bees. Anim Behav.

[CR8] Breed MD, Leger EA, Pearce AN, Wang YJ (1998). Comb wax effects on the ontogeny of honey bee nestmate recognition. Anim Behav.

[CR9] Breed MD, Perry S, Bjostad LB (2004). Testing the blank slate hypothesis: why honey bee colonies accept young bees. Insect Soc.

[CR10] Breed DB, Cook CN, McCreery HF, Rodriguez M, Aquiloni L, Tricarico E (2015). Nestmate Recognition in Eusocial Insects: The Honeybee as a Model System. Social recognition in invertebrates.

[CR11] Châline N, Sandoz JC, Martin SJ, Ratnieks FLW, Jones GR (2005). Learning and discrimination of individual cuticular hydrocarbons by honey bees (*Apis mellifera*). Chem Senses.

[CR12] Crailsheim K, Stolberg E (1989). Influence of diet, age and colony condition upon intestinal proteolytic activity and size of the hypopharyngeal glands in the honeybee (*Apis mellifera* L.). J Insect Physiol.

[CR13] Cruz-Landim C, Ferreira-Caliman MJ, Gracioli-Vitti JF, Zucchi R (2012). Correlation between mandibular gland secretion and cuticular hydrocarbons in the stingless bee *Melipona quadrifasciata*. Genet Mol Res.

[CR14] Dani FR, Morgan ED, Turillazzi S (1996). Dufour gland secretion of *Polistes* wasps: chemical composition and possible involvement in nestmate recognition (Hymenoptera: Vespidae). J Insect Physiol.

[CR15] Dani FR, Jones GR, Corsi S, Beard R, Pradella D, Turillazzi S (2005). Nest mate recognition cues in the honey bee: differential importance of cuticular alkanes and alkenes. Chem Senses.

[CR16] Decio P, Vieira AS, Dias NB, Palma MS, Bueno OC (2016). The Postpharyngeal gland: specialized organ for lipid nutrition in leaf-cutting ants. PLoS One.

[CR17] Downs SG, Ratnieks FLW (1999). Recognition of conspecifics by honey bee guards (*Apis mellifera*) uses non-heritable cues applied in the adult stage. Anim Behav.

[CR18] Duffield RM, Wheeler JW, Eickwort GC, Bell WJ, Cardé RT (1984). Sociochemicals of bees. Chemical ecology of insects.

[CR19] Free JB (1987). Pheromones of social bees.

[CR20] Fröhlich B, Riederer M, Tautz J (2000). Comb-wax discrimination by honeybees tested with the proboscis extension reflex. J Exp Biol.

[CR21] Fujita T, Kozuka-Hata H, Uno Y, Nishikori K, Morioka M, Oyama M, Kubo T (2010). Functional analysis of the honeybee (*Apis mellifera* L.) salivary system using proteomics. Biochem Biophys Res Commun.

[CR22] Guillem RM, Drijfhout FP, Martin SJ (2016). Species-specific cuticular hydrocarbon stability within European *Myrmica* ants. J Chem Ecol.

[CR23] Herznera G, Rutherb J, Gollerc S, Schulzc S, Goettlera W, Strohma E (2011). Structure, chemical composition and putative function of the postpharyngeal gland of the emerald cockroach wasp, *Ampulex compressa* (Hymenoptera, Ampulicidae). Zool.

[CR24] Howard RW, Baker JE (2003). Morphology and chemistry of Dufour glands in four ectoparasitoids: *Cephalonomia tarsalis, C. waterstoni* (Hymenoptera: Bethylidae), *Anisopteromalus calandrae*, and *Pteromalus cerealellae* (Hymenoptera: Pteromalidae). Comp Biochem Physiol B.

[CR25] Huang ZY, Otis GM, Teal PEA (1989). Nature of brood signal activating of protein synthesis of hypopharyngeal gland in honey bees, *Apis mellifera* (Apidae: Hymenoptera). Apidologie.

[CR26] Kaib M, Eisermann B, Schoeters E, Billen J, Franke S, Francke W (2000). Task-related variation of postpharyngeal and cuticular hydrocarbon compositions in the ant *Myrmicaria eumenoides*. J Comp Physiol A.

[CR27] Kalmus H, Ribbands CR (1952). The origin of the odours by which honeybees distinguish their companions. Proc R Soc Lond B.

[CR28] Kather R, Martin SJ (2015). Evolution of Cuticular hydrocarbons in the Hymenoptera: a meta-analysis. J Chem Ecol.

[CR29] Kather R, Drijfhout FP, Martin SJ (2011). Task group differences in cuticular lipids in the honey bee *Apis mellifera*. J Chem Ecol.

[CR30] Katzav-Gozansky T, Soroker V, Ionescu A, Robinson GE, Hefetz A (2001). Task-related chemical analysis of labial gland volatile secretion in worker honeybees (*Apis mellifera ligustica*). J Chem Ecol.

[CR31] Kurstjens SP, Hepburn HR, Schoening FRL, Davidson BC (1985). The conversion of wax scales into comb wax by African honeybees. J Comp Physiol B.

[CR32] Le Conte Y, Hefetz A (2008). Primer Pheromones in Social Hymenoptera. Ann Rev Entomol.

[CR33] Le Conte Y, Mohammedi A, Robinson GE (2001). Primer effects of a brood pheromone on honeybee behavioural development. Proc R Soc Lond B.

[CR34] Martin SJ, Drijfhout FP (2009). How reliable is the analysis of complex cuticular hydrocarbon profiles by multi-variate statistical methods?. J Chem Ecol.

[CR35] Martin SJ, Helanterä H, Drijfhout FP (2008). Colony-specific hydrocarbons identify nest mates in two species of *Formica* ant. J Chem Ecol.

[CR36] Martin SJ, Weihao Z, Drijfhout FP (2009). Long-term stability of cuticular hydrocarbons facilitates chemotaxonomy. Biol J Linn Soc.

[CR37] Maurizio A, Crane E (1975). How bees make honey. Honey a comprehensive survey.

[CR38] McCune B, Grace JB (2002) Analysis of ecological communities. MjM Software Design: Gleneden Beach, Oregon, USA, p 300

[CR39] Meskali M, Bonavita-Cougourdan A, Provost E, Bagnères AG, Dusticier G, Clement J-L (1995). Mechanism underlying cuticular hydrocarbon homogeneity in the ant *Camponotus vagus* (Scop.) (Hymenoptera: Formicidae): role of postpharyngeal glands. J Chem Ecol.

[CR40] Mitra A (2013). Function of the Dufour’s gland in solitary and social Hymenoptera. J Hymenopt Res.

[CR41] Mitra A, Gadagkar R (2014). Dufour’s gland and the cuticle in the social wasp *Ropalidia marginata* contain the hydrocarbons in similar proportions. J Insect Sci.

[CR42] Mitteroecker P, Bookstein F (2011). Linear discrimination, ordination, and the visualization of selection gradients in modern Morphometrics. Evol Biol.

[CR43] Morgan ED (2010). Biosynthesis in insects: advanced edition.

[CR44] Nelson DR, Tissot M, Nelson LJ, Fatland CL, Gordon DM (2001). Novel wax esters and hydrocarbons in the cuticular surface lipids of the red harvester ant, *Pogonomyrmex barbatus*. Comp Biochem Physiol B.

[CR45] Niño EL, Malka O, Hefetz A, Tarpy DR, Grozinger CM (2013). Chemical profiles of two pheromone glands are differentially regulated by distinct mating factors in honey bee queens (*Apis mellifera* L.). PLoS One.

[CR46] Oldham N, Billen J, Morgan ED (1994). On the similarity of the Dufour gland secretion and the cuticular hydrocarbons of some bumblebees. Physiol Entomol.

[CR47] Page RE, Metcalf RA, Metcalf RL, Erickson EH, Lampman RL (1991). Extractable hydrocarbons and kin recognition in honey bee (*Apis mellifera* L.). J Chem Ecol.

[CR48] Pitts-Singer TL, Hagen MM, Helm BR, Highland S, Buckner JS, Kemp WP (2017). Comparison of the chemical compositions of the cuticle and dufour’s gland of two solitary bee species from laboratory and field conditions. J Chem Ecol.

[CR49] Plettner E, Otis GW, Wimalaratne PDC, Winston ML, Slessor KN, Pankiw T, Punchihewa PWK (1997). Species and caste determined mandibular gland signals in honeybees (*Apis*). J Chem Ecol.

[CR50] Poiani SB, Cruz-Landim CD (2010). Morphological changes in the cephalic salivary glands of females and males of *Apis mellifera* and *Scaptotrigona postica* (Hymenoptera, Apidae). J Biosci.

[CR51] Poiani SB, Cruz-Landim C (2017). Comparison and correlation between chemical profiles of the cephalic salivary glands and cuticle surface of workers of *Apis mellifera* (Hymenoptera, Apidae). Can J Zool.

[CR52] Pradella D, Martin SJ, Dani FR (2015). Using errors by guard honeybees (*Apis mellifera*) to gain new insights into nestmate recognition signals. Chem Senses.

[CR53] Simpson J (1960). The functions of the salivary glands of *Apis mellifera*. J Insect Physiol.

[CR54] Simpson J (1963). The source of the saliva honeybees used to moisten materials they chew with their mandibles. J Apic Res.

[CR55] Snodgrass RE (1956) Anatomy of the honey bee. Comstock Publishing Associates; Cornell university Press, Ithica

[CR56] Šobotnik J, Kalinova B, Cahlikova L, Weyda F, Ptaček V, Valterova I (2008). Age-dependent changes in structure and function of the male labial gland in *Bombus terrestris*. J Insect Physiol.

[CR57] Soroker V, Vienne C, Nowbahari E, Hefetz A (1994). The post-pharyngeal gland as a “gestalt” organ for nestmate recognition in the ant *Cataglyphis niger*. Naturwissenschaften.

[CR58] Sutton P, Wilde MJ, Martin SJ, Cvacka J, Vrkoslav V, Rowland SJ (2013). Studies of long chain lipids in insects by (HT) GC and HTGC-MS. J Chromatogr A.

[CR59] Tofilski A (2012) Honey bee. Available from http://www.honeybee.drawwing.org. Accessed 15 May 2016

[CR60] Vallet A, Cassier P, Lensky Y (1991). Ontongeny of the fine structure of the mandibular glands of the honeybees (*Apis mellifera* L.) workers and the pheromonal activity of 2-heptanone. J Insect Physiol.

[CR61] van Zweden JS, d’Ettorre P (2010) Nestmate recognition in social insects and the role of hydrocarbons. Pp 222–243 In: Blomquist GJ, Bagnères AG (eds). Insect hydrocarbons: Biology, Biochemistry and Chemical Ecology. Cambridge University Press

[CR62] Waś E, Szczęsna T, Rybak-Chmielewska H (2014). Determination of beeswax hydrocarbons by gas chromatography with a mass detector (GC-MS) technique. J Apic Sci.

[CR63] Wyatt TD (2013) Pheromones and animal behaviour: communication by smell and taste. 2^nd^ edn. Cambridge University Press

